# Felcisetrag stimulates 5-HT_4_-serotonin receptors in the human atrium

**DOI:** 10.1007/s00210-025-04606-w

**Published:** 2025-09-29

**Authors:** Lina Maria Rayo Abella, Joachim Neumann, Britt Hofmann, Uwe Kirchhefer, Ulrich Gergs

**Affiliations:** 1 Institute for Pharmacology and Toxicology, Medical Faculty, Martin-Luther-University Halle-Wittenberg, Magdeburger Straße 4, D-06097 Halle (Saale), Germany; 2 Department of Cardiac Surgery, Mid-German Heart Centre, University Hospital Halle, Ernst-Grube Straße 40, D-06097 Halle (Saale), Germany; 3 Institute for Pharmacology and Toxicology, Medical Faculty, University Münster, Domagkstraße 12, D-48149 Munster, Germany

**Keywords:** Felcisetrag, Human atrium, Mouse atrium, 5-HT_4_-serotonin receptors, Transgenic mice

## Abstract

Felcisetrag (methyl 4-[[4-[[(2-propan-2-yl-1*H*-benzimidazole-4-carbonyl)-amino]-methyl]-piperidin-1-yl]methyl]piperidine-1-carboxylate, TD-8954, TAK-954) has a structural formula with similarity to serotonin. It is one of the most potent compounds to bind to recombinant human 5-HT_4_-serotonin receptors. We noted that felcisetrag raised force of contraction in left atrial preparations (LA) and beating rate in right atrial preparations (RA) from mice with cardiac-specific overexpression of the human 5-HT_4_ receptors (5-HT_4_-TG) but was inactive in LA and RA from adult wild type mouse hearts (WT). When felcisetrag had increased force of contraction in LA or beating rate in RA of 5-HT_4_-TG, GR125487, a 5-HT_4_ receptor antagonist, reduced force of contraction and beating rate. Felcisetrag raised force of contraction only in the presence of cilostamide in human right atrial preparations (HAP) obtained from adult patients during open heart surgery due to severe coronary heart disease. These positive inotropic effects of felcisetrag in HAP were attenuated by 1 µM GR125487. In the presence of cilostamide, 100 nM felcisetrag exerted positive inotropic effects that were increased further by 1 µM serotonin. When 1 µM serotonin had raised force of contraction, additionally applied 100 nM felcisetrag reduced force of contraction in HAP. These data suggest that felcisetrag can act as an agonist as well as an antagonist at human 5-HT_4_ receptors in the mammalian heart.

## Introduction

**Fig. 1:**
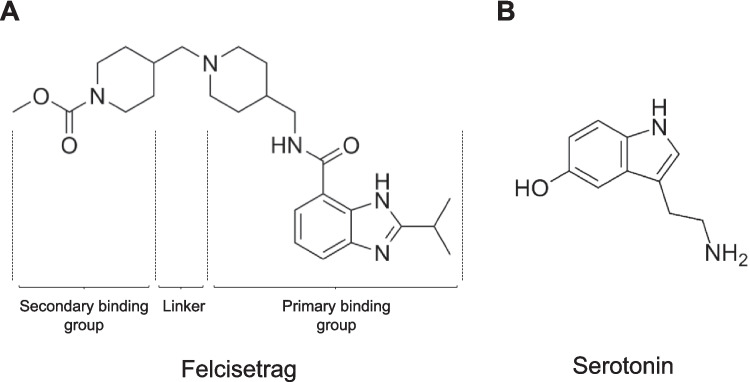
Structural formulae of felcisetrag and serotonin. **A** Felcisetrag can be seen as a derivative of **B** serotonin (5-hydroxytryptamine, 5-HT). This might explain its potency and selectivity

Felcisetrag (Fig. 1A: methyl 4-[[4-[[(2-propan-2-yl-1*H*-benzimidazole-4-carbonyl)-amino]methyl]piperidin-1-yl]methyl]piperidine-1-carboxylate) can be structurally regarded as a derivative of serotonin (5-HT, Fig. 1B) and is an experimental drug that has gastrointestinal prokinetic properties (Beattie et al. 2011). Felcisetrag acts as a potent (pK_i_-value = 9.3) ligand at recombinant 5-HT_4_ serotonin receptors (Beattie et al. 2011). Moreover, felcisetrag potently stimulates the gastrointestinal tract of animals and humans (Beattie et al. 2013). Hence, in the future, felcisetrag might become clinically relevant. Felcisetrag was developed by a company called Theravance Inc., in San Francisco, California, USA, led by a multivalent design approach (McKinnell et al. 2013). It consists of a primary binding group, a linker, and a secondary binding group (Fig. 1A) and is a partial agonist with respect to cAMP generation in assays with 5-HT_4_-receptor–transfected cell lines (McKinnell et al. 2013).

We decided to study the cardiac effects of felcisetrag because it is hitherto the most potent agonist at human 5-HT_4_ receptors with a rang order of potency of felcisetrag > tegaserod > prucalopride > mosapride (Beattie et al. 2011). Moreover, felcisetrag was also reported as a very selective agonist for 5-HT_4_ receptors because felcisetrag showed little binding to other receptors (Beattie et al. 2011). In the past, we and others have studied the cardiac effects of these other compounds similar to felcisetrag (cisapride: e.g., Kaumann et al. 1991; Bach et al. 2001; Keller et al. 2018; prucalopride: Keller et al. 2018; tegaserod: e.g., Hesse et al. 2024; mosapride: Neumann et al. 2024a). To the best of our knowledge, the possibility that felcisetrag might stimulate or block 5-HT_4_ receptors in the human heart has hitherto not been explored.

Why may cardiac 5-HT_4_ receptors be of clinical relevance? For one, 5-HT_4_ receptors exist and are functional in the human heart via stimulation of adenylyl cyclases (Dumuis et al. 1989). In the isolated muscle strips or cardiomyocytes from right and left human auricles, these 5-HT_4_ receptors augment the current through the L-type calcium cation channels and thereby directly elevate the force of cardiac contraction (Kaumann and Levy 2006; Ouadid et al. 1992; Jahnel et al. 1993). Interestingly, 5-HT_4_ receptor agonists raised force only in the failing but not the nonfailing human ventricle (Brattelid et al. 2004). Only pig and monkey atria, but not atria from rat, rabbit, dog, or normal mouse, display 5-HT_4_ receptor-mediated increases in beating rate and force of contraction (Medhurst and Kaumann 1993, review: Neumann et al. 2023). To have a small animal model for the human atrium, we have generated and characterized in past, transgenic mice with overexpression of functional human 5-HT_4_ receptors not only in cardiomyocytes of the atrium but also in the ventricle (5-HT_4_-TG: Gergs et al. 2010, 2013). In the past, we have detected and quantified the expression of human 5-HT_4_ receptors in 5-HT_4_-TG and noted much higher expression than in human atrium (Gergs et al. 2009, 2017; Neumann et al. 2021b; Hesse et al. 2024). Thence, we have utilized the 5-HT_4_-TG to detect even small cardiac effects of agonists at 5-HT_4_ receptors like the approved prokinetic drugs prucalopride, cisapride, metoclopramide, tegaserod, mosapride, bromopride, and clebopride in left and right atrial preparations (Keller et al. 2018; Neumann et al. 2021a, 2024a; Hesse et al. 2024; Abella et al. 2025a, b). This preparatory work motivated us now to study in 5-HT_4_-TG also the cardiac effects of the investigational drug felcisetrag for comparison with our published studies. Therefore, we hypothesized that felcisetrag not only binds to human 5-HT_4_ receptors in the test tube but also acts as a partial agonist to stimulate and inhibit human cardiac function by acting directly on cardiomyocytes rather than via central nervous mechanisms.

It is unlikely that felcisetrag will ever be used to treat heart failure, atrial fibrillation, hypertension, or cardiac sepsis, diseases in which serotonin certainly or possibly plays a role (review: Neumann et al. 2023). However, 5-HT_4_ receptor agonists and 5-HT_4_ receptor antagonists may in the future play a role for non-cardiac diseases, e.g., in the treatment of obesity, depression, and Morbus Alzheimer by acting on brain 5-HT_4_ receptors. Moreover, 5-HT_4_ receptor agonists are already used in the clinic to ameliorate some gastrointestinal diseases like reflux in the esophagus or gastroparesis (Beattie et al. 2011). Hence, a broad spectrum of indications for felcisetrag is conceivable. A prime advantage of felcisetrag compared to its predecessors lies in its higher affinity and higher selectivity for the human 5-HT_4_ receptors (Beattie et al. 2011). Cardiac side effects of felcisetrag might also gain important relevance from a drug safety aspect. For instance, while cisapride or tegaserod are quite useful to treat gastrointestinal diseases, they have lethal cardiovascular side effects that have limited their usefulness or even led to their removal from the drug market (Giudicessi et al. 2018; Lacy et al. 2022). As the objective of this study was to elucidate the mechanism of felcisetrag in the human heart, human right atrial preparations (HAP) were utilized as a model for the human heart in vivo. To the best of our knowledge, the contractile effects of felcisetrag have not been studied in atria from 5-HT_4_-TG or in HAP.

In summary, we tested the following three hypotheses:

1. Felcisetrag amplifies force of contraction in isolated left atrial preparations from 5-HT_4_-TG via 5-HT_4_ receptors.
2. Felcisetrag raises the beating rate in spontaneously beating right atrial preparations from 5-HT_4_-TG via 5-HT_4_ receptors.
3. Felcisetrag augments force of contraction in HAP via 5-HT_4_ receptors.

## Materials and methods

### Transgenic mice

Mice in this study included transgenic mice (CD1 background) where the full-length human 5-HT_4_ receptor is overexpressed in the heart driven by α-myosin heavy chain promoter (5-HT_4_-TG). The generation and initial characterization of these mice on a biochemical and functional level has been reported some years ago (Gergs et al. 2010). For comparison, we used littermate wild type animals (WT). Mice were aged about 150 days and of random gender. The investigation conformed to the Guide for the Care and Use of Laboratory Animals as published by the National Research Council (2011). The animals were handled and maintained according to the approved protocols of the Animal Welfare Committee of the University of Halle-Wittenberg, Halle, Germany (permission: I8M9).

In brief, the right or left atrial preparations from the mice were isolated and mounted in organ baths as previously described (Gergs et al. 2013; Neumann et al. 1998). The bathing solution (a modified Tyrode’s solution) of the organ baths contained 119.8 mM NaCI, 5.4 mM KCI, 1.8 mM CaCl_2_, 1.05 mM MgCl_2_, 0.42 mM NaH_2_PO_4_, 22.6 mM NaHCO_3_, 0.05 mM Na_2_EDTA, 0.28 mM ascorbic acid, and 5.05 mM glucose. Ascorbic acid was always present to inhibit the oxidation and thus the inactivation of isoprenaline and serotonin. The solution was continuously gassed with 95% O_2_ and 5% CO_2_ and maintained at 37 °C and pH 7.4 (Neumann et al. 1998). Force of contraction was quantified in vertically mounted, electrically paced isolated left atrial preparations and in spontaneously beating right atrial preparations under isometric conditions. The duration of electrical stimulation with a rectangular impulse of direct current lasted for 5 ms. The voltage was 10% higher than necessary to initiate contraction. Muscles were stretched such that the maximum basal force was generated and then allowed to stabilize for 30 min before drug application started. Spontaneously beating right atrial preparations from mice were used to study any chronotropic effects. The drug application was as follows. After equilibration was reached following three changes of the Tyrode’s solution in the organ baths (10 ml volume), felcisetrag was cumulatively or non-cumulatively added to left atrial or right atrial preparations to establish concentration- and time-response relationships. In some experiments, serotonin and isoprenaline or 5-HT_4_ receptor antagonists were added (see figure legends for details).

### Contractile studies on human preparations

The contractile studies on human preparations were done using the same setup and the same modified Tyrode’s solution as used in the mouse studies. In brief, force of contraction was quantified in electrically paced isolated right atrial preparations under isometric conditions. The duration of electrical stimulation with a rectangular impulse of direct current lasted for 5 ms. The voltage was 10% higher than necessary to initiate contraction. Muscles were stretched such that the maximum basal force was generated and then allowed to stabilize for 30 min with three changes of the buffer in the organ baths before drug application started. The samples were obtained from 15 male and 5 female patients, aged 54–83 years. The patients suffered from coronary diseases (two and three vessel diseases). Cardiac comorbidities included atrial arrhythmias, chronic heart failure, and hypertension. Cardiac drug therapy included acetyl salicylic acid, apixaban, furosemide or other diuretics, metoprolol or other β-adrenoceptor antagonists, and statins. Our methods used for atrial contraction studies in human samples have been previously published and were not altered over the years for this study (e.g., Gergs et al. 2009; Abella et al. 2025a). The drug application was as follows. After equilibration was reached, felcisetrag was cumulatively or non-cumulatively added to human right atrial preparations to establish concentration- and time-response curves. The next half logarithmic higher concentration was added as soon as force of contraction had reached a new plateau. In separate experiments, first cilostamide was given. We waited until a small positive inotropic effect to cilostamide had developed and reached a plateau. We used here as in the past cilostamide because it is a potent inhibitor of phosphodiesterase III which is the main phosphodiesterase in the human heart (e.g., Neumann et al. 2024b). Cilostamide does not increase force of contraction in the mouse atrium because the mouse heart is regulated mainly via phosphodiesterase IV which is not inhibited by cilostamide but by, e.g., rolipram (e.g., Neumann et al. 2019). In some preparations, also a 5-HT_4_ receptor antagonist was added (GR 125487) or serotonin or isoprenaline was applied; for details, see original recordings and legends. The study in patients complies with the Declaration of Helsinki and has been approved by the local Ethics Committee of the Medical Faculty of the Martin Luther University Halle-Wittenberg, Halle, Germany (hm-bü).

### Data analysis

Data shown are means ± standard error of the mean. Statistical significance was estimated using the analysis of variance followed by Bonferroni’s *T*-test or the Student’s *T*-test as appropriate. A *p*-value < 0.05 was considered to be significant. A commercial software was used to quantify force of contraction, its first derivative with respect to time, and time parameters like time to peak tension or time to relaxation (LabChart 8 from AD Instruments, Oxford, England, and GraphPad Prism 9, California, USA).

### Drugs and materials

The drugs isoprenaline-hydrochloride (Merck, Dreieich, Germany), serotonin hydrochloride (Merck, Dreieich, Germany), felcisetrag (MedChemExpress via Hycultec, Beutelsbach, Germany), GR 125487 (5-fluoro-2-methoxy-[1-[2-[(methylsulfonyl)amino]ethyl]−4-piperidinyl]−1*H*-indole-3-methylcarboxylate sulfamate (Tocris via Bio-Techne, Wiesbaden, Germany) (Claeysen et al. 2000; Nirogi et al. 2016), and cilostamide (N-cyclohexyl-N-methyl-4-(1,2-dihydro-2-oxo-6-quinolyloxy)butyramide, Merck, Dreieich, Germany) were used. All other chemicals were of the highest purity grade commercially available. Deionized water was used throughout the experiments for preparation of the modified Tyrode’s solution. Stock solutions were prepared fresh daily.

## Results

**Fig. 2:**
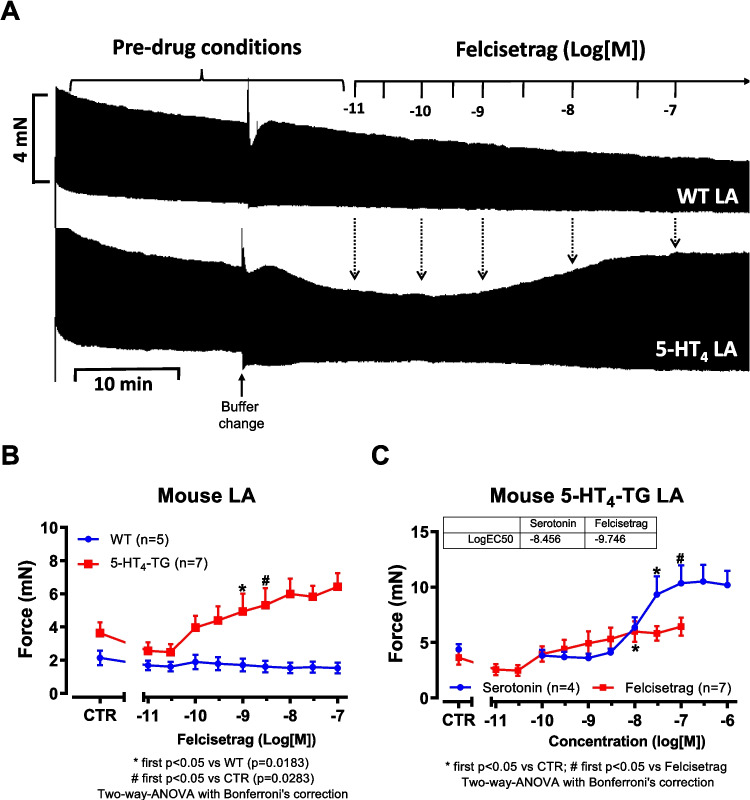
Felcisetrag increases force of contraction in LA from 5-HT_4_-TG. **A** Original recording in mouse left atrial preparation from WT (top) or from 5-HT_4_-TG (bottom). Felcisetrag was cumulatively applied and induced a time- and concentration-dependent positive inotropic effect in 5-HT_4_-TG. **B** Summarized concentration-response curves for the effect of felcisetrag on force of contraction in milli Newton (mN). **C** Comparison of the positive inotropic effect of felcisetrag to the positive inotropic effect of serotonin. Numbers in brackets mean number of mice. CTR (= control) indicates pre-drug value

**Fig. 3:**
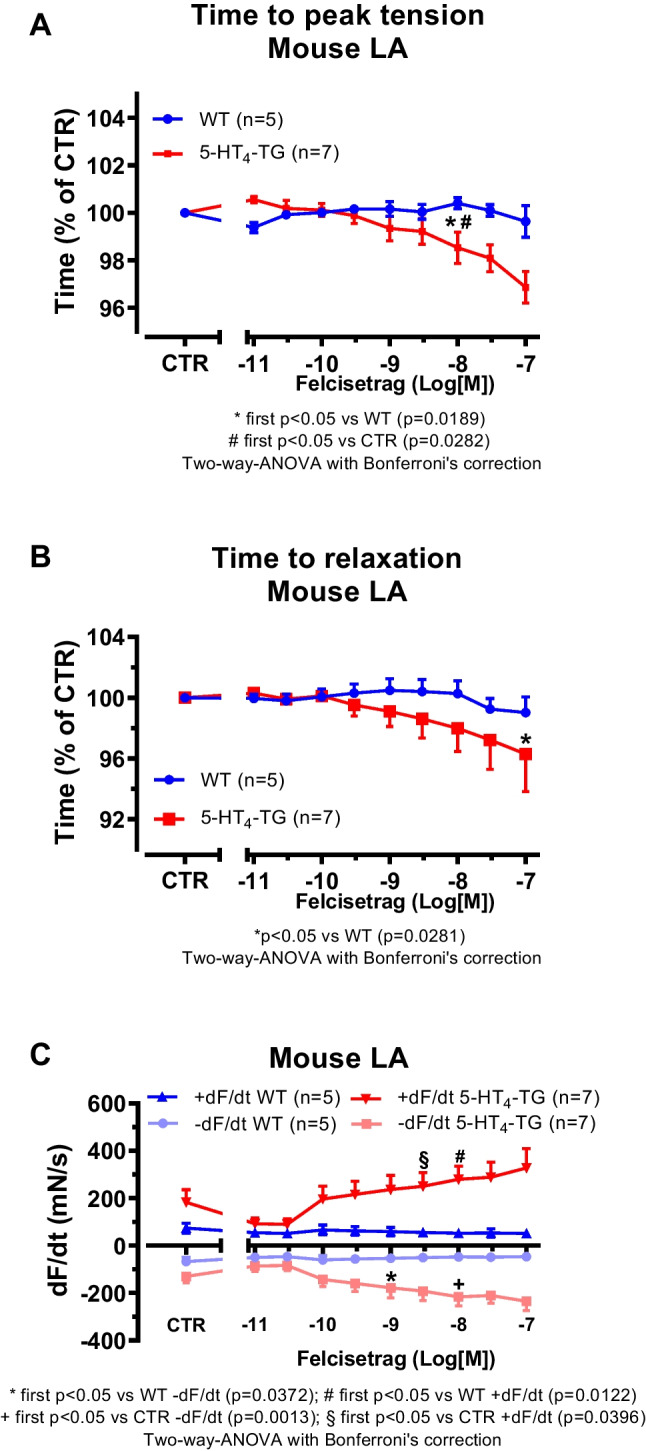
Felcisetrag affects contractile parameters in LA from 5-HT_4_-TG. **A** Summarized data for the concentration-dependent effect of cumulatively applied felcisetrag in left atrial preparations from 5-HT_4_-TG for time to peak tension in percentage of CTR (pre-drug values) and **B** time to relaxation in percentage of CTR, **C** rate of tension development (dF/dt_max_) and rate of tension relaxation (dF/dt_min_), both in mN per seconds (mN/s). Numbers in brackets mean number of mice. CTR (= control) indicates pre-drug value

Initially, mouse atrial preparations were studied, and based on these findings, the research was extended to human atrial preparations in the present study. Thus, cumulatively applied felcisetrag exerted a concentration- and time-dependent positive inotropic effects in left atrial preparations from 5-HT_4_-TG. This is depicted in an original recording in Fig. 2A. These positive inotropic effects started at 3 nM and reached a maximum at 100 nM (the highest concentration studied). A direct comparison of the inotropic effects of felcisetrag and serotonin in 5-HT_4_-TG LA demonstrated a higher potency but lower efficacy of felcisetrag compared to serotonin (Fig. 2C). In contrast, under the same experimental conditions (performed on the same experimental day with littermates in adjacent organ baths), felcisetrag failed to raise force of contraction in left atrial preparations from WT (Fig. 2A). The latter finding is in agreement with our previous work: 5-HT cannot raise force in atrium from WT (Gergs et al. 2010). The expression or coupling of the 5-HT_4_ receptor is supposedly too small to affect contractility (discussed in Gergs et al. 2013). Moreover, we were interested in the effect of felcisetrag on time of contraction. This was done because cAMP-dependent pathways should shorten time to peak tension via phosphorylation of the ryanodine receptor, and this would release more Ca^2+^ per unit time from the sarcoplasmic reticulum (cf. Gergs et al. 2009, 2010). Moreover, cAMP-dependent pathways should reduce the time to relaxation because phospholamban phosphorylation will activate SERCA, more Ca^2+^ per unit time is removed from the vicinity of myofilament, and the heart relaxes. Similarly, as we reported for serotonin in our previous reports (Gergs et al. 2013), also felcisetrag reduced time to peak tension (Fig. 3A) and shortened the time to relaxation in preparations from 5-HT_4_-TG but not from WT (Fig. 3B, Gergs et al. 2010). Felcisetrag started at 1 nM or at 3 nM to increase the rate of relaxation and the rate of tension development, respectively (Fig. 3C).

**Fig. 4:**
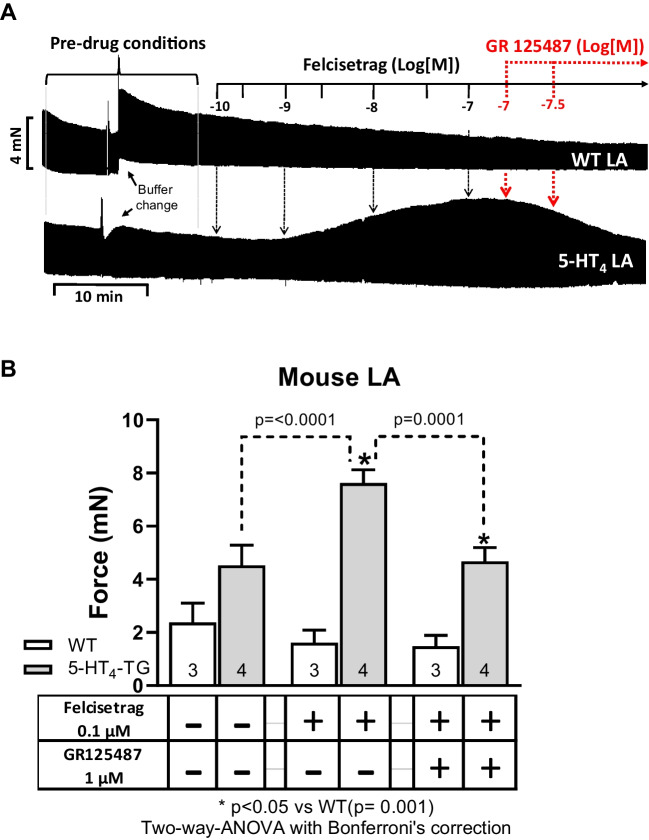
GR 125487 reversed the positive inotropic effects of felcisetrag in LA from 5-HT_4_-TG. **A** Original recording in mouse left atrial preparations from WT (top) and 5-HT_4_-TG (bottom). Each drug addition is indicated by a vertical arrow. Felcisetrag induced a time- and concentration-dependent positive inotropic effect in 5-HT_4_-TG, which was reversed by additionally applied GR 125487. **B** Summarized effects of felcisetrag alone or in the additional presence of GR 125487 on force of contraction in mN. Numbers in bars mean numbers of mice. CTR (= control) indicates pre-drug value (left bars)

To corroborate the view that felcisetrag acts via 5-HT_4_ receptors, we studied next whether GR 125487, a 5-HT_4_ receptor antagonist, would reverse the positive inotropic effect of felcisetrag in the left atrium of 5-HT_4_-TG. This was the case. First, this is visualized in an original tracing (Fig. 4A). In left atrial preparations from WT, felcisetrag did not increase force of contraction (Fig. 4A, top). Additionally applied increasing concentrations of GR 125487 diminished the force of contraction in left atrial preparations from 5-HT_4_-TG (Fig. 4A, bottom). Several experiments such as in Fig. 4A are summarized in the bar diagram in Fig. 4B.

**Fig. 5:**
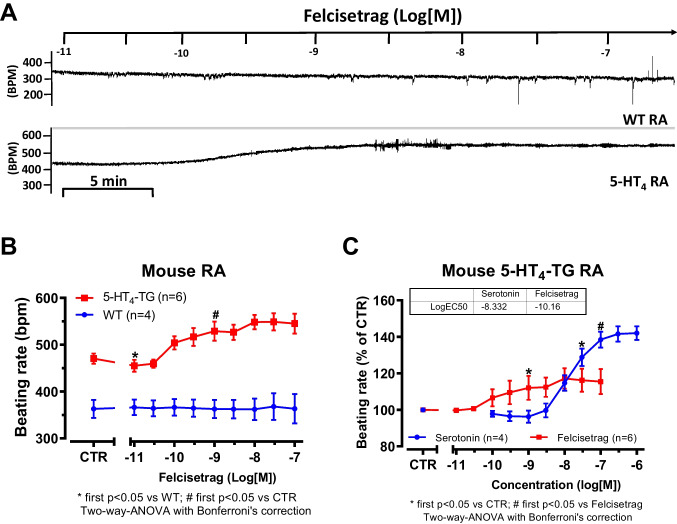
Felcisetrag increased the beating rate in RA from 5-HT_4_-TG. **A** Original recording in mouse right atrial preparations. Felcisetrag induced a time- and concentration-dependent positive chronotropic effect in 5-HT_4_-TG (bottom) but not in WT (top). **B** Summarized concentration-response curve for the effect of felcisetrag on beating rate in 5-HT_4_-TG (squares) but not in WT (circles). **C** Comparison of the positive chronotropic effect of felcisetrag to the positive inotropic effect of serotonin normalized to the control (CTR). Numbers in brackets mean number of mice. CTR indicates pre-drug value

**Fig. 6:**
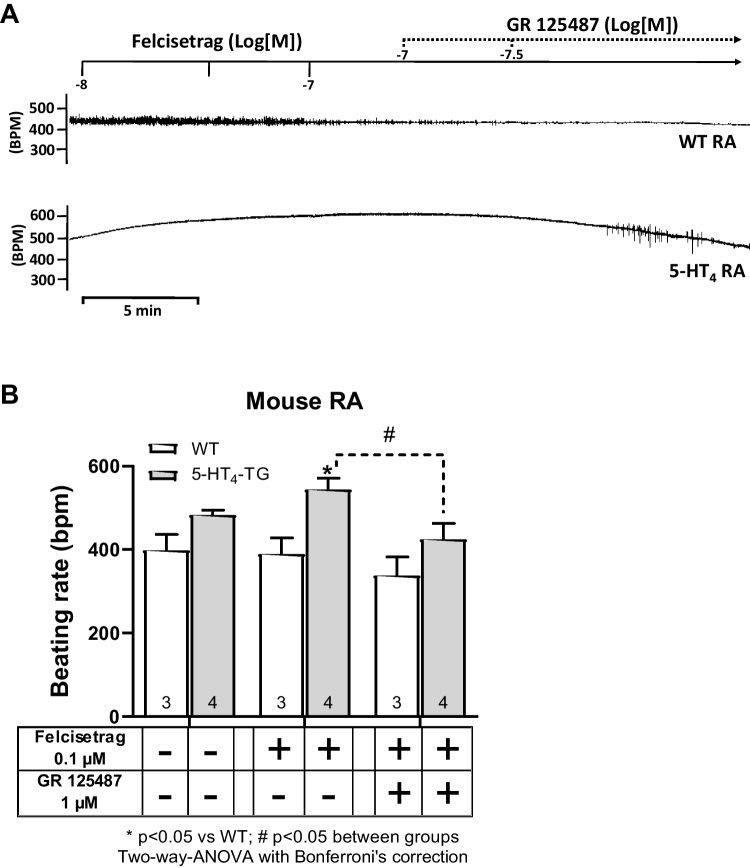
GR 125487 reversed the positive chronotropic effects of felcisetrag in RA from 5-HT_4_-TG. **A** Original recording in mouse right atrial preparations. Felcisetrag (Felci) induced a time- and concentration-dependent positive chronotropic effect in 5-HT_4_-TG (bottom) but not in WT (top). Subsequently, GR 125486 (GR) was applied. The positive chronotropic effect of felcisetrag in 5-HT_4_-TG that is antagonized by GR125486 (bottom). **B** Bar diagrams indicate the beating rate of WT or 5-HT_4_-TG RA in the presence of felcisetrag alone or in the additional presence of GR 125487. Numbers in brackets mean number of mice. CTR (= control) indicates pre-drug value (left bars)

If felcisetrag acted like serotonin, felcisetrag should augment the beating rate in the right atrium of 5-HT_4_-TG but not in WT. Indeed, we noticed a time- and concentration-dependent positive chronotropic effect of felcisetrag only in 5-HT_4_-TG and not in right atrial preparations from WT. That is plotted in Fig. 5A in an original recording. Mean data are shown in Fig. 5B. Here, it is noteworthy that already 1 nM felcisetrag elevated the beating rate and that higher concentrations were not more effective (Fig. 5B). In contrast, the positive chronotropic effect started a 3 nM serotonin in right atrial preparations from 5-HT_4_-TG and higher concentrations of serotonin were of increasing efficacy (Gergs et al. 2013). A direct comparison of the chronotropic effects of felcisetrag and serotonin in 5-HT_4_-TG RA demonstrated a higher potency but lower efficacy of felcisetrag compared to serotonin (Fig. 5C). This indicates a partial agonistic effect of felcisetrag on beating rate in this transgenic model. These effects of felcisetrag on the beating rate in right atrial preparations from 5-HT_4_-TG were antagonized by GR 125487 as seen in an original recording in Fig. 6A. Mean data on beating rate by felcisetrag are depicted in Fig. 6B. These data confirm that felcisetrag acts via 5-HT_4_ receptors to augment beating rate.

**Fig. 7:**
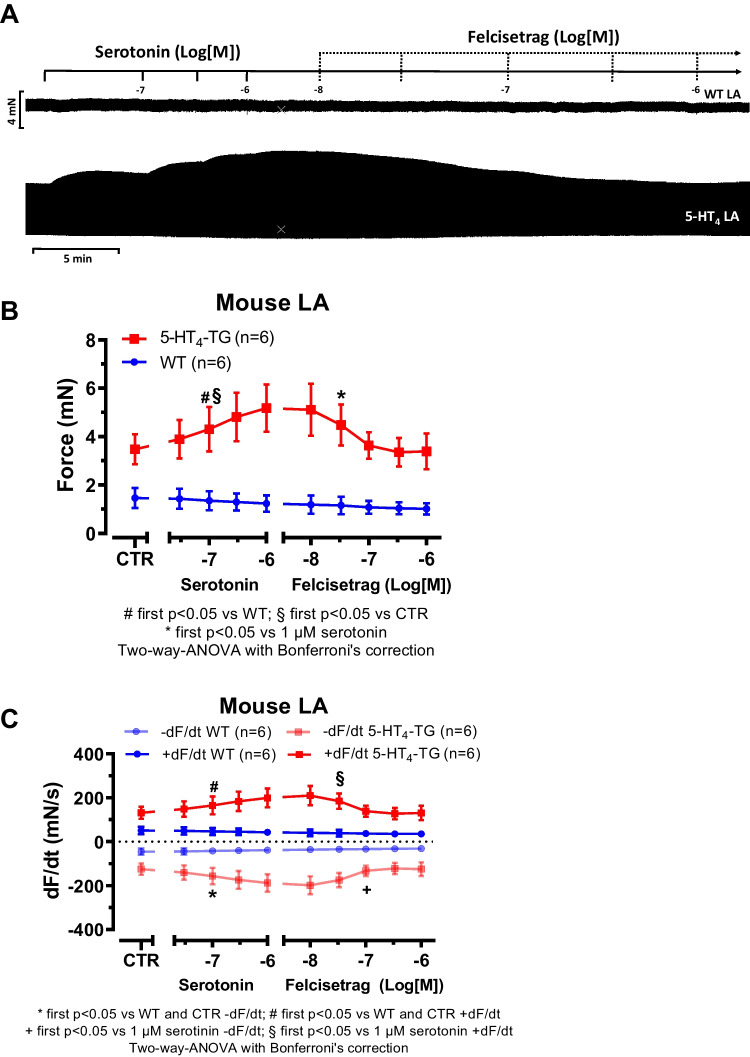

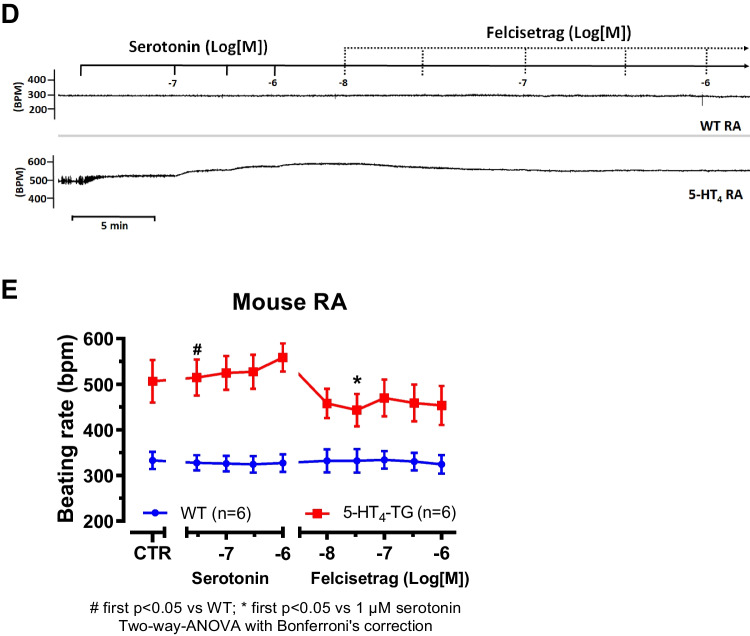
Felcisetrag reduced the positive inotropic effect of serotonin in LA and the positive chronotropic effect of serotonin in RA from 5-HT_4_-TG. **A** Original recording of the concentration- and time-dependent positive inotropic effect of serotonin and then increasing concentrations of felcisetrag in LA from 5-HT_4_-TG. **B** Summarized effect of serotonin alone and after felcisetrag on force of contraction in milli Newton (mN) in LA from 5-HT_4_-TG. **C** Summarized effect of serotonin alone and after felcisetrag on the rate of tension development (dF/dt_max_) and rate of tension relaxation (dF/dt_min_) in mN/s. **D** Original recording of the concentration- and time-dependent positive chronotropic effect of serotonin and then increasing concentrations of felcisetrag in RA from 5-HT_4_-TG. **E** Summarized effect of serotonin alone and after felcisetrag on the beating rate in beats per minute (bpm) in RA from 5-HT_4_-TG. Numbers in brackets mean number of mice. CTR (= control) indicates pre-drug value

We had shown repeatedly that 5-HT_4_ receptor agonists, used in gastroenterology, are often partial and not full agonists at cardiac 5-HT_4_ receptors (e.g., Hesse et al. 2024). Using a protocol as published in past reports, we assessed this possibility also here. Indeed, when the force of contraction was increased with 1 µM serotonin in left atrial preparations from 5-HT_4_-TG followed by the application of increasing concentrations of felcisetrag, the force of contraction was reduced. This is seen in an original recording in Fig. 7A. Mean data are shown in Fig. 7B for force of contraction. Moreover, the same pattern emerged when we plotted the first derivative of force versus time (Fig. 7C). We often report not only measurements of force but also of the first derivative because it is generally thought that the derivative is a more physiological and sensitive measure of contractility than the developed force (e.g., Braunwald et al. 1967).

Moreover, we wanted to know whether felcisetrag also acts as a partial agonist for the beating rate. To this end, we first increased beating rate with serotonin in right atrial preparations from 5-HT_4_-TG, followed by the application of increasing concentrations of felcisetrag (Fig. 7D). In these preparations, felcisetrag reduced beating rate (Fig. 7D, E). In WT preparations, there was no chronotropic effect of serotonin, nor of felcisetrag (Fig. 7D, E).

**Fig. 8:**
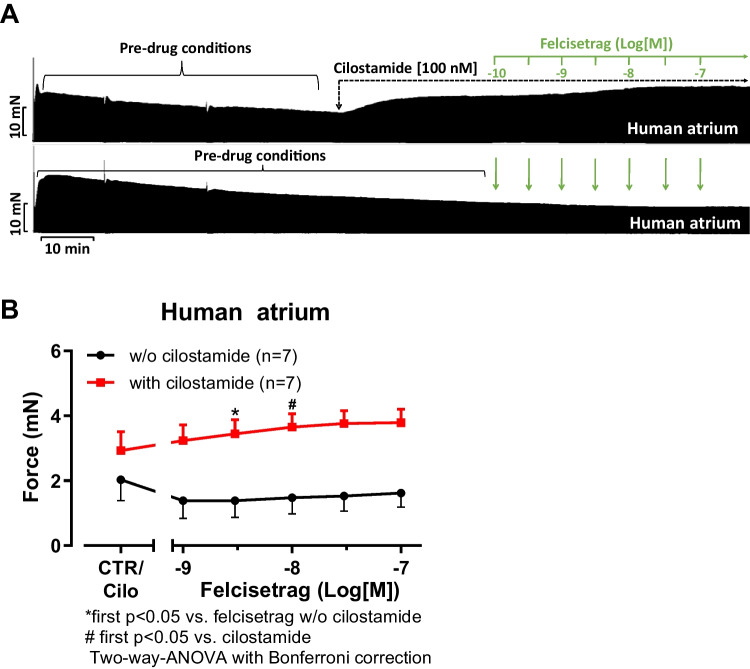

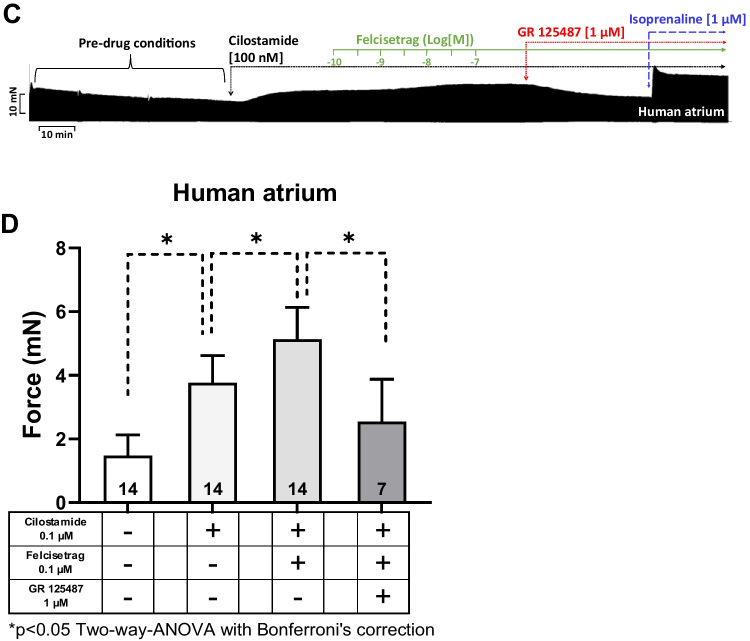
Felcisetrag increased force of contraction in HAP. **A** Original recordings of force of contraction in the presence (top) or absence (bottom) of cilostamide (Cilo) and additionally applied felcisetrag in milli Newton (mN) in electrically stimulated human right atrial muscle strips. Each drug addition is indicated by a vertical arrow. Only after cilostamide, felcisetrag induced a concentration- and time-dependent positive inotropic effect in HAP. **B** Summarized effects of felcisetrag in the presence (squares) and absence (circles) of cilostamide in milli Newton (mN) in electrically stimulated human right atrial muscle strips. CTR: force without cilostamide before addition of felcisetrag. Cilo: force with cilostamide before addition of felcisetrag. **C** Original recording of force of contraction in the presence of cilostamide and additionally applied felcisetrag followed by GR 125487 (GR) and isoprenaline in milli Newton (mN) in electrically stimulated human right atrial muscle strips. Each drug addition is indicated by a vertical arrow. **D** Summarized effects of cilostamide alone, felcisetrag in the presence of cilostamide and additionally applied GR 125487 on force of contraction in electrically stimulated human right atrial muscle strips. Numbers in brackets mean number of muscle strips from seven patients

**Fig. 9:**
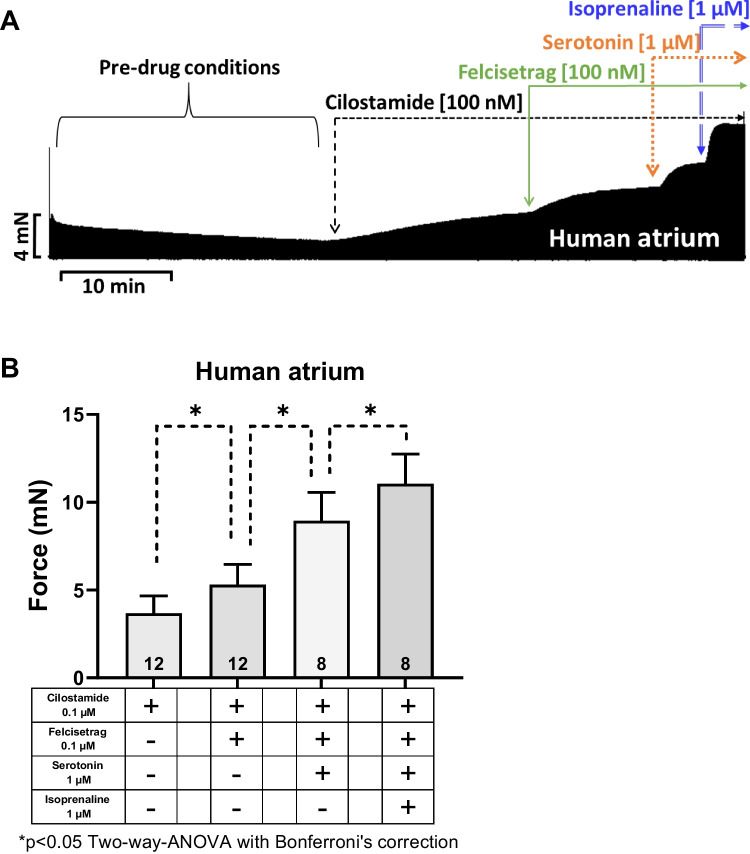

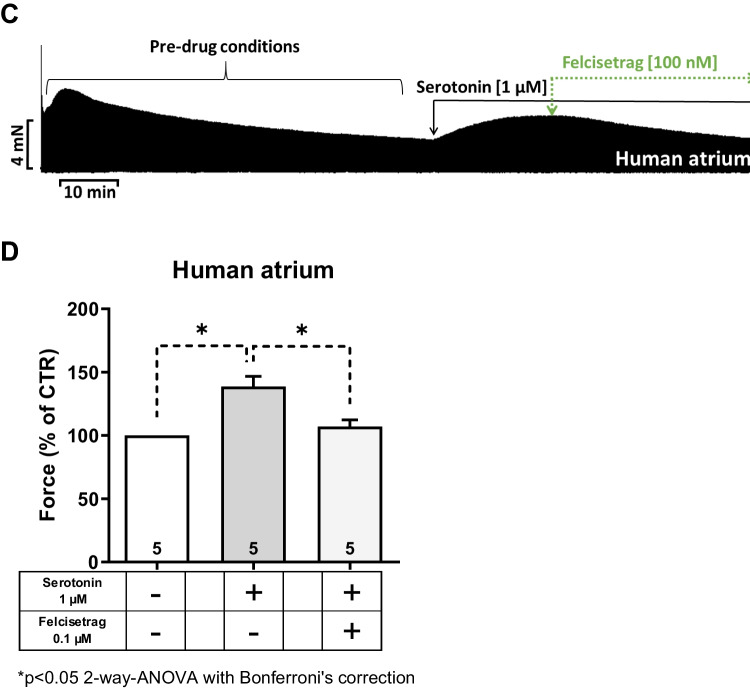
Felcisetrag is a partial agonist in HAP. **A** Original recording of force of contraction of cilostamide and additionally applied felcisetrag and thereafter serotonin followed by isoprenaline in milli Newton (mN) in electrically stimulated human right atrial muscle strips. Each drug addition is indicated by a vertical arrow. **B** Bar diagram summarizing the effects of cilostamide alone and additionally applied felcisetrag, serotonin, and isoprenaline on force of contraction in HAP. **C** Original recording of force of contraction with serotonin and additionally applied felcisetrag in milli Newton (mN) in electrically stimulated human right atrial muscle strips. **D** Bar diagram summarizing the effects of serotonin alone and felcisetrag after serotonin on force of contraction in percentage of CTR (= control, pre-drug value, left bar) in HAP. Numbers in brackets mean number of muscle strips from five to eight patients

Next, we wanted to test the effects of felcisetrag in the human heart. We used the same methods, which we had used repeatedly for the study of 5-HT_4_ serotonin agonists in HAP in the past (e.g., Neumann et al. 2021a; Hesse et al. 2024). Therefore, we mounted human atrial preparations in the organ bath, stimulated them electrically, and obtained concentration response curves for felcisetrag. As seen in the original recording in Fig. 8A, felcisetrag alone failed to raise the force of contraction. This is in stark contrast with our findings in atria from 5-HT_4_-TG (Fig. 2). That does not mean that felcisetrag does not act on 5-HT_4_ receptors in HAP, as will be shown in the subsequent figure. Indeed, and in agreement with our work on other 5-HT_4_ receptor agonists in HAP (e.g., Abella et al. 2025a), when we first slightly stimulated force by inhibiting phosphodiesterase III by use of cilostamide, we observed a time-dependent and concentration-dependent increase force of contraction in HAP. This is seen in an original recording (Fig. 8A). Several similar experiments on force of contraction are summarized in Fig. 8B. Please keep note that at 10 nM felcisetrag, this increase in force of contraction gained significance (Fig. 8B). However, the possibility exists that alternative receptors, such as adrenergic receptors or the release of noradrenaline, may underlie the positive inotropic effects of felcisetrag in HAP. But this possibility appears to be improbable. Indeed, when in separate experiments, we first gave cilostamide and then felcisetrag and then added GR 125487 would reduce force of contraction (Fig. 3C). A minor side observation was that this negative inotropic effect of GR 125487 was not due to an irreversible damage of the HAP by GR125487: isoprenaline could markedly raise force of contraction (Fig. 8C for an original tracing). Mean data are shown and tested statistically in Fig. 8D. Hence, we suggest that felcisetrag acts via 5-HT_4_ receptors in HAP. Like in the left atrial preparations of 5-HT_4_-TG, the question arose, how effective is felcisetrag to raise force of contraction? This was addressed in a similar manner as in several recent reports from our group in HAP (e.g., Neumann et al. 2024a). We first gave cilostamide. This raised force of contraction in HAP (Fig. 9A). Then, we added a single high concentration of felcisetrag. This augmented force of contraction in HAP further (Fig. 9A). Then, a high concentration of serotonin was able to elevate force of contraction even more (Fig. 9A). However, serotonin was less effective than isoprenaline to augment force of contraction in HAP. A well-known observation first reported from Kaumann’s group (Kaumann et al. 1990) was that isoprenaline was more effective than serotonin to elevate force of contraction in HAP (Fig. 9A). To be able to perform statistical tests and ascertain this finding, we repeated such experiments several times and obtained Fig. 9B. Drugs that are not full receptor agonists are often partial agonists at these receptors, at which they can also act antagonistically, as can be seen with the atrial preparations in Fig. 7 or in our earlier reports (e.g., Hesse et al. 2024). Indeed, when we first elevated force of contraction by a high concentration of 1 µM serotonin (which is maximum for the positive inotropic effects in HAP and left atrial preparations, Kaumann et al. 1990; Gergs et al. 2009), then additional felcisetrag reduced force of contraction in HAP. This can be seen in a typical recording in Fig. 9C and is summarized in Fig. 9D. Hence, felcisetrag is not a full agonist but a partial agonist at HAP with respect to force of contraction.

## Discussion

### Main new finding

The main, clinically relevant, finding of this communication is that felcisetrag can act as an agonist at 5-HT_4_ receptors in isolated human cardiac preparations to raise force of contraction.

### Mechanism of felcisetrag

The positive inotropic effects started at 3 nM felcisetrag in the present study in left atrial preparations from 5-HT_4_-TG. These effects were reversed by GR 125487, a 5-HT_4_ receptor antagonist. Moreover, inotropic effects of felcisetrag were lacking in left atrial preparations from WT. These two data sets concur that the inotropic effects of felcisetrag are mediated by 5-HT_4_ receptors in 5-HT_4_-TG. However, felcisetrag was more potent to increase force of contraction in 5-HT_4_-TG than in HAP. We noted this discrepancy in previous studies on drugs like metoclopramide or clebopride and assume this is due to the higher level of expression of the 5-HT_4_ receptor in 5-HT_4_-TG compared to HAP (Neumann et al. 2021a). Likewise, as concerns efficacy, felcisetrag is more effective in left atrial preparations from 5-HT_4_-TG than in HAP. We have made this observation repeatedly with, e.g., metoclopramide or tegaserod (Neumann et al. 2021a; Hesse et al. 2024). We explain this apparent discrepancy also with a higher expression of 5-HT_4_ receptors in 5-HT_4_-TG than in HAP. Moreover, when we first gave serotonin to maximally increase force of contraction, then additionally applied felcisetrag reduced force of contraction in left atrial preparations from 5-HT_4_-TG or in HAP. This might be explained if we assumed that felcisetrag under these conditions acts not only as an agonist but also as an antagonist at cardiac 5-HT_4_ receptors.

When we compare the mouse data with the human data, we noted that it is likely that felcisetrag also in HAP acts via 5-HT_4_ receptors. We can conclude from this that felcisetrag is only a partial, less efficacious agonist than serotonin at cardiac 5-HT_4_ receptors and therefore application of felcisetrag post-serotonin appears to have an antagonistic effect. When we stimulated the receptor in HAP with 1 µM serotonin, then felcisetrag reduced force of contraction. Comparable results have already been observed in other cases, for example, with mosapride, tegaserod, bromopride, and clebopride in HAP (e.g., Hesse et al.; Abella et al. 2025a, b). Moreover, when felcisetrag stimulated maximal force of contraction in the HAP, then additionally applied serotonin could increase force of contraction further. We noted similar results before, e.g., with clebopride (Abella et al. 2025a). These findings are consistent with the interpretation that felcisetrag is a partial agonist in HAP as in left atrial preparations from 5-HT_4_-TG and in line with our previous reports on other prokinetic drugs (e.g., Neumann et al. 2024a). These data are consistent with binding data generated in recombinant expression systems, in which the binding affinity of felcisetrag to human 5-HT_4(c)_ receptors was reported as follows: pK_i_ = 9.4 and pEC_50_ = 9.3, and the intrinsic activity was 83% of the maximum activity of serotonin (Beattie et al. 2011).

Our findings in HAP are somewhat different from our animal studies: first of all, felcisetrag only increased force of contraction in HAP in the presence of a phosphodiesterase inhibitor. Moreover, felcisetrag increased force of contraction less potently in HAP than in left atrial preparations from 5-HT_4_-TG. These receptors in HAP are functionally blocked by a 5-HT_4_ receptor antagonist (GR 125487), as this antagonist reduced the force of contraction during felcisetrag application. Similar as with bromopride or clebopride in HAP, we have had to add cilostamide to detect a positive inotropic effect of felcisetrag. In contrast, in left atrial preparations from 5-HT_4_-TG, felcisetrag, bromopride, or clebopride alone increased force of contraction (Abella et al. 2025a, b). This argues that felcisetrag is a similarly effective agonist as bromopride or clebopride at least in HAP. However, felcisetrag is more potent than all other 5-HT_4_ receptor agonists we used before in 5-HT_4_-TG or HAP. Please also consider that felcisetrag is much more potent to raise force of contraction in left atrial preparations and in HAP than, for instance, bromopride or clebopride. This is made plausible by the fact that they belong to different chemical classes: benzimidazole derivative (felcisetrag) versus benzamide derivatives (bromopride, clebopride). In addition, felcisetrag was less effective to raise force of contraction than serotonin in HAP or left atrial preparations when compared to our own previous studies with serotonin alone (Gergs et al. 2009, 2010). Under our experimental conditions, metoclopramide, bromopride, and clebopride likewise reduced 5-HT-stimulated force of contraction and were therefore regarded not as pure agonists but also as partial agonists (Abella et al. 2025a; Neumann et al. 2021a): thus, by comparison with drugs we studied previously, we regard felcisetrag also as a partial agonist at 5-HT_4_ receptors in HAP. The fact that phosphodiesterase inhibition by cilostamide (indeed, we only detected a positive inotropic effect of felcisetrag in the presence of cilostamide in HAP) potentiated the positive inotropic effects of felcisetrag is consistent with the known fact that serotonin via 5-HT_4_ receptors increased cAMP in HAP (Kaumann et al. 1990; Gergs et al. 2009). This assumption that cAMP is involved in the contractile effects of felcisetrag in HAP is also supported by the observation that felcisetrag increased the rate of muscle relaxation and shortened the time to relaxation in HAP. These effects also occur with isoprenaline or serotonin and are accompanied and explained by an increased phosphorylation of phospholamban due to elevated cAMP or more precisely by activation of the cAMP-dependent protein kinase and subsequent phosphorylation of phospholamban at amino acid serine 16 (Gergs et al. 2009). The observation that isoprenaline is more effective to raise force of contraction than serotonin in HAP is long known and is explained by the higher density of β-adrenoceptors compared to 5-HT_4_ receptors in the HAP (Kaumann et al. 1990). This is not true in left atrial preparations from 5-HT_4_-TG: in these left atrial preparations, serotonin and isoprenaline are equieffective, which we explained by a higher expression of 5-HT_4_ receptors in 5-HT_4_-TG than in HAP with regard to β-adrenoceptors (Gergs et al. 2010, 2013; Neumann et al. 2021b).

Felcisetrag is chemically very similar to velusetrag being also a benzimidazole like felcisetrag but produced by a different drug company. We have recently noted in a preliminary form that velusetrag like felcisetrag potently increased force of contraction in 5-HT_4_-TG, suggesting a potent class effect of benzimidazoles in these cardiac preparations (Schmidt et al. 2025).

### Effects on beating rate

Please note that like serotonin (Gergs et al. 2013) also felcisetrag stimulated 5-HT_4_ receptors in the sinus node of transgenic mouse heart (5-HT_4_-TG). This conclusion is based on the observation that any positive chronotropic effects of felcisetrag are absent in right atrial preparations from WT, and that these positive chronotropic effects are present in 5-HT_4_-TG. Moreover, the positive chronotropic effects of felcisetrag were antagonized by GR 125487, a 5-HT_4_ receptor antagonist. The felcisetrag acted like various other agonist in our hands (cisapride, prucalopride, metoclopramide) and like them as a less effective agonist compared to the effect of 5-HT: the positive chronotropic effect was smaller than those of serotonin in previous report under the same experimental conditions (Gergs et al. 2013).

Our data cannot prove that the same mechanisms for a positive chronotropic effect hold true in the human heart. However, we think it is likely that at least qualitatively similar effects are expected to occur in the human sinus node for two reasons. The human sinus node contains functionally 5-HT_4_ receptors, and intravenous infusion of serotonin causes a positive chronotropic effect in humans (LeMessurier et al 1959). This can also be speculated from electrophysiological data measured in myocytes isolated from human right atrial tissue using 5-HT_4_ receptor agonists which were blocked by 5-HT_4_ receptor antagonists (Pino et al. 1998). Moreover, we have overexpressed the human 5-HT_4_ receptors in the transgenic mice and did find with 5-HT_4_ receptor agonists and 5-HT_4_ receptor antagonists that they behave as expected with respect to beating rate in isolated left atrial preparations but also in isolated perfused hearts and in vivo using telemetric recording of the electrocardiogram (e.g., Gergs et al. 2010, 2013). It must be mentioned, however, that to our knowledge, there are no data on cells of the sinus node in humans.

### Clinical relevance

We find currently eight finished or ongoing clinical trials in www.clinical trials.gov for TAK-954 or TD-8954, the internal company names for felcisetrag. For instance, one study tried to improve with felcisetrag the gastrointestinal propulsion in patients after gut surgery (Boeckxstaens et al. 2024). Felcisetrag was beneficial in patients that suffered from gastroparesis (Chedid et al. 2021).

In humans, felcisetrag has a half-life of about 70 h (Pusalkar et al. 2022). In therapeutic dosage, a peak plasma concentration of about 7 nM was recorded (Czerniak et al. 2022). Please note that this is about the concentration of felcisetrag where we detect a positive inotropic effects in HAP. Felcisetrag is mainly eliminated in humans by the kidney and is metabolized in the liver via the cytochrome CYP3A4 (Chen et al. 2022). Hence, one can understand why inhibitors of CYP3A4 like itraconazole were found to inhibit the metabolism of felcisetrag and why itraconazole was noticed to increase the plasma concentration of felcisetrag (Chen et al. 2022; Pusalkar et al. 2022). Since felcisetrag is predominantly eliminated via the kidneys, it is possible that plasma levels of felcisetrag may be elevated in patients with kidney disease, potentially causing increased cardiac side effects. However, due to the limited data available on a drug that has not yet been approved, this is only a speculation. Nevertheless, such data must be collected in the future.

Interestingly, 5-HT_4_ receptor agonists may be useful to treat depression and Morbus Alzheimer (Lanthier et al. 2020). Indeed, we recently reported on such an investigational drug (Neumann et al. 2024c). Hence, in Morbus Alzheimer, felcisetrag might be studied in humans, and this is supported by animal studies of felcisetrag (Shen et al. 2011). From that view also, a knowledge of cardiac side effects of felcisetrag is clinically relevant.

It is accepted that 5-HT_4_ receptors are present in human atrium. This is clear from ligand binding studies and from contraction experiments performed by Kaumann’s group (Kaumann et al. 1990). Moreover, electrophysiological studies noted the functional presence of 5-HT_4_ receptors in human atrium (Neumann et al. 2023 for overview). Hence, we can predict that felcisetrag can stimulate in principle human atrial 5-HT_4_ receptors in patients. This receptor stimulation might lead to a beneficial positive inotropic effect. However, this effect should be limited to the left and right atrium and thus will have only a limited contribution to the overall cardiac performance, because in the non-failing heart, serotonin is ineffective (unless a phosphodiesterase inhibitor is added) to raise force of contraction in the human ventricle (Brattelid et al. 2004). Admittedly, if a patient has left atrial fibrillation, less blood is pumped into the left ventricle, and in this sense, the left atrium might be clinically relevant in these patients. However, in patients suffering from severe systolic heart failure, the density of 5-HT_4_ receptors was increased, at least at the mRNA level (Brattelid et al. 2004). To the best of our knowledge, the protein expression of the 5-HT_4_ receptors in the failing and nonfailing human ventricle has not yet been compared, but here we speculate that protein expression of 5-HT_4_ receptor might be enhanced in heart failure. This would explain why in ventricular muscle preparations of these heart failure patients, serotonin was able to increase force of contraction (Brattelid et al. 2004). Thus, felcisetrag might increase ventricular force of contraction but only in heart failure patients. A caveat on felcisetrag is in order. Stimulation of cardiac 5-HT_4_ receptors can lead to arrhythmias and as such would be detrimental (Kaumann 1994; Keller et al. 2018). In general, all cAMP-increasing agents have the potential to cause arrhythmias, even though the exact mechanism may vary (Tamargo et al. 2011; Tisdale et al. 2020; Fischer et al. 2015; Potenza et al. 2019). However, felcisetrag has the advantage over other prokinetic drugs that did not lead to vasoconstriction of isolated human coronary arteries in contrast to serotonin itself (Beattie et al. 2011).

### Limitations of the study

We have in all our samples a decrease in force of time (run-down). To this end, we are showing longer recordings to make this visible. However, this run-down does not diminish the interpretation of our data. Indeed, we decided not to correct for any run-down, because thus we may underestimate but we do not overestimate the positive inotropic effect of felcisetrag. So the positive inotropic effect of felcisetrag is probably larger than presented here. However, by refraining for a run-down correction (which can also be criticized as having an arbitrary aspect as the run-down may not be linear or constant over time), we can be very confident to have reported the lower limit of the positive inotropic effect of felcisetrag which may lead to further studies. One could further argue that we noted a positive inotropic effect of felcisetrag only in the presence of a phosphodiesterase inhibitor. However, we would counter that some patients may take phosphodiesterase inhibitors like levosimendan, pimobendane, or theophylline (Fitton and Brogden 1994; Tilley 2011; Orstavik et al. 2014; Rayo Abella et al. 2023a, b). Although this is purely speculative at present, because felcisetrag has not yet been approved and thus wide experience in patients is lacking, we would expect felcisetrag to have a positive inotropic effect in such patients. Another limitation of our study is that we did not investigate human ventricular muscle strips. However, we currently have no access to that tissue. Based on the work of others, we would predict that in the presence of a phosphodiesterase inhibitor, felcisetrag should increase force of contraction human ventricles from failing and non-failing hearts because others reported that in the presence of the unspecific phosphodiesterase inhibitor 3-isobutyl-1-methylxanthine (better known as IBMX), serotonin raised force of contraction in all ventricular human muscle strips tested: i.e., in ventricular muscle strips from failing and non-failing hearts (Brattelid et al. 2004).

In summary, we can now affirm the hypotheses raised in the “Introduction” in this way:

1. Felcisetrag amplifies force of contraction in isolated left atrial preparations from 5-HT_4_-TG via 5-HT_4_ receptors.
2. Felcisetrag raises the beating rate in spontaneously beating right atrial preparations from 5-HT_4_-TG via 5-HT_4_ receptors.
3. Felcisetrag augments force of contraction in the presence of a phosphodiesterase inhibitor in HAP via 5-HT_4_ receptors.

## Data Availability

No datasets were generated or analysed during the current study.
